# Colchicine Binding Site Tubulin Inhibitors Impair Vincristine-Resistant Neuroblastoma Cell Function

**DOI:** 10.3390/molecules30102186

**Published:** 2025-05-16

**Authors:** Cinthia N. Reed, Kaylee B. Garrison, Joshua Thammathong, Jindrich Cinatl, Martin Michaelis, Souvik Banerjee, April M. Weissmiller

**Affiliations:** 1 Department of Biology, Middle Tennessee State University, Murfreesboro, TN 37132, USA; cnr3h@mtmail.mtsu.edu (C.N.R.); kaylee.chisam1255@gmail.com (K.B.G.); 2 Department of Chemistry, Middle Tennessee State University, Murfreesboro, TN 37132, USA; jt8p@mtmail.mtsu.edu (J.T.); souvik.banerjee@mtsu.edu (S.B.); 3 Dr Petra Joh Research Institute, Frankfurter Stiftung für Krebskranke Kinder, 60528 Frankfurt am Main, Germany; j.cinatl@kinderkrebsstiftung-frankfurt.de (J.C.J.); m.michaelis@kent.ac.uk (M.M.); 4 School of Natural Sciences, University of Kent, Canterbury CT2 7NJ, UK

**Keywords:** *MYCN*, *ALK*, neuroblastoma, vincristine, colchicine binding site inhibitor, tubulin

## Abstract

High-risk neuroblastoma remains a clinically challenging pediatric cancer, with an approximate five-year survival rate of ~60%. Frontline therapy for this group of patients includes surgery and intensive chemotherapy that involves combinations of the tubulin inhibitor vincristine with several other chemotherapeutics. Unfortunately, unresponsiveness to therapy and relapse are common, with tumors often displaying resistance to vincristine. Recently, we characterized a novel set of tubulin inhibitors that are distinct from vincristine and bind within the colchicine binding site present on tubulin monomers. Colchicine binding site inhibitors (CBSIs) have gained traction as improved chemotherapeutics due to their potential to overcome tubulin inhibitor-induced resistance. In this study, we investigate the functional impact of CBSI treatment on multiple neuroblastoma cell lines, including those that are vincristine-resistant. We demonstrate that our newly developed compounds are effective at disrupting cell division in non-resistant and resistant cells and have cellular activity against vincristine-resistant cell lines. Interestingly, we find that vincristine-resistant cell lines differ in their ability to undergo apoptotic cell death in response to CBSI treatment. Taken together, these findings provide a solid foundation to further investigate the utility of CBSIs for neuroblastoma treatment, while highlighting the distinct resistance mechanisms that can emerge in these childhood cancers.

## 1. Introduction

Neuroblastoma is the most common extracranial tumor found in infants, with ~40% of all cases diagnosed before the age of one [1]. Clinically, patients are classified as either low-, intermediate-, or high-risk based on factors such as the extent of disease, molecular pathological features, and age [1,2]. While upwards of 90% of low- and intermediate-risk patients survive, children with high-risk diseases have poor overall survival (~60%) despite aggressive multimodal therapies that include surgery, chemotherapy, radiation, stem cell transplant, immunotherapy, and differentiation therapy [1,2]. The first phase of treatment for high-risk patients, which is known as induction therapy, involves surgery and multiple rounds of intensive chemotherapy that combines tubulin inhibitors (i.e., vincristine), topoisomerase inhibitors (i.e., etoposide, doxorubicin), alkylators (i.e., cyclophosphamide), and platinum compounds (i.e., cisplatin, carboplatin). Current regimens, and those in ongoing clinical trials, use varying combinations of these chemotherapeutic agents and always include vincristine [2].

Response to induction therapy predicts disease outcome, with patients who show poor response having the most dismal prognosis [2]. Resistance can be due to neuroblastoma cells acquiring drug resistance during chemotherapy or already having a primary resistance to compounds at the initiation of treatment [1]. Tumors can become resistant to drug treatment through multiple mechanisms, with the most direct mechanism being increased expression of the ATP-binding cassette (*ABC*) family of transporters that function to remove drugs from the cell. The major three *ABC* transporter genes implicated in cancer drug resistance are *ABCB1*, *ABCC1*, and *ABCG2*. In neuroblastoma, resistance is linked to multiple genetic and epigenetic changes and is not solely derived from overexpression of *ABC* transporters, although expression of some, such as *ABCC4*, is strongly associated with poor patient outcome [1,3]. Overall, the complexity of resistance mechanisms results in approximately half of patients being unresponsive to induction therapy or showing cancer relapse within two years [1], indicating that resistance to chemotherapy continues to be a barrier to alleviating high-risk diseases.

Recently, we discovered a series of novel small-molecule tubulin polymerization inhibitors that target the colchicine binding site located at the interface of the α- and β-heterodimer [4], a region of action distinct from inhibitors of the taxane or vinca alkaloid sites [5]. Inhibitors of the latter subtypes, namely paclitaxel for taxanes and vincristine or vinblastine for vinca alkaloids, are commonly used to treat a wide array of malignancies in the clinic, even though their effectiveness is restricted by the emergence of multi-drug resistance and toxicities [6,7]. The development of CBSIs as improved tubulin inhibitors has gained traction over the last decade due to their decreased susceptibility to drug resistance mechanisms and in vivo efficacy against multiple cancers [8,9,10]. As vinca alkaloid tubulin inhibitors such as vincristine are included in frontline neuroblastoma therapy—and resistance to vincristine is present clinically in recurrent neuroblastoma [11]—the development of CBSIs could have therapeutic value for these tumors. Indeed, clinical trials in children with relapsed and/or refractory neuroblastoma testing the efficacy of an early-generation colchicine binding site inhibitor, ABT-751, indicate that CBSI treatment can increase the duration of event-free survival [11]. These results are encouraging, especially when considering the potency of CBSIs has substantially improved since the discovery of ABT-751. Thus, CBSIs shows potential as new and more effective chemotherapies than current first-in-line tubulin inhibitors.

However, as of now the functional impact of CBSI treatment on neuroblastoma cells is not clear, and it is also unknown if any newly developed CBSIs are effective against vincristine-resistant neuroblastoma cells. Given the high heterogeneity of neuroblastoma cell lines that mirrors the diversity of tumors and outcomes for patients with these cancers [12,13,14], clarifying the impact of CBSI action on multiple cell lines may provide insight into their value as novel anticancer chemotherapeutics. Therefore, in this study, we investigate the impact of CBSI treatment on neuroblastoma cell function using two novel CBSI compounds identified as lead compounds based on previous molecular modeling, binding studies, cellular efficacy, and biological evaluation. These two compounds, named 4h and 4k, each have an imidazo [1,2-a]pyrazine core, although changes in ring D substituents confer 4h with higher cellular potency but lower metabolic stability than 4k [4]. We find through employing these two CBSIs that both compounds induce mitotic protein marker expression in treated neuroblastoma cells, which is consistent with their ability to block mitosis. We also find that 4h can impair colony formation, with lower inhibition correlating with *MYCN*-amplification status. Importantly, in neuroblastoma cell lines that are resistant to vincristine, 4h and 4k impair cellular viability, disrupt intracellular microtubule networks, induce mitotic protein marker expression, and promote the accumulation of cells within the G2/M phase of the cell cycle. However, only 4h can induce apoptosis in all non-resistant neuroblastoma cells, including the vincristine-resistant *MYCN*-amplified cell line, indicating that 4h is more active across all biological assays tested when compared to 4k. The results of this study suggest that newly developed CBSIs may offer value as therapeutics for neuroblastoma, particularly CBSIs like 4h that have high cellular potency. Furthermore, this research highlights differences in the ability of CBSIs to induce apoptosis when cells have become vincristine resistant, suggesting that further work should be focused on resistant cell lines to develop more efficacious CBSIs.

## 2. Results

### 2.1. Impact of Colchicine Binding Site Inhibition on Neuroblastoma Cell Lines

#### 2.1.1. Cell Viability Paneling Using Diverse Neuroblastoma Cells

**Figure 1:**
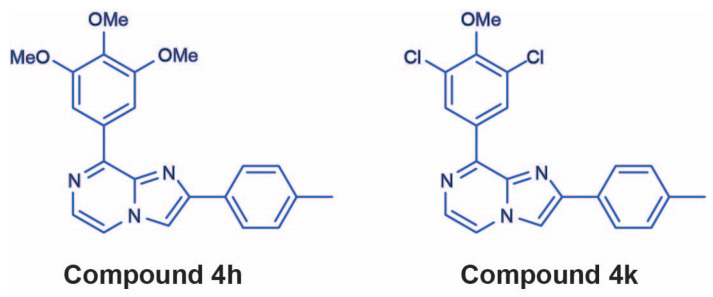
Chemical structures of 4h and 4k, which were identified and evaluated as colchicine binding site inhibitors in [4].

Previously, we paneled the cellular activity of 4h and 4k (Figure 1) against three *MYCN*-amplified neuroblastoma cell lines: CHP-134, Kelly, and Be(2)C [4]. These cell lines were chosen for our initial study because *MYCN* amplification is present in ~50% of high-risk neuroblastoma cases [15,16]. However, dysregulation of the *MYC* family member that encodes c-MYC is also implicated in high-risk cases, and anaplastic lymphoma kinase (*ALK*) mutations occur in up to 14% of high-risk tumors [17,18,19]. Therefore, to understand how 4h and 4k broadly influence neuroblastoma cell function, we paneled the cellular activity of both compounds against four *MYCN* single copy lines, SH-EP, SH-SY5Y, SK-N-AS, and SK-N-SH, which each differ in their genetic risk factors. We also paneled both compounds against an additional *MYCN*-amplified IMR-32 line and included an assessment of CHP-134 and Kelly cells as a comparison to our previously published data. In line with our original findings [4], in CHP-134 and Kelly cells, 4h is more potent than 4k at decreasing cell viability (Table 1). IMR-32 cells were impacted similarly with growth inhibition of 50% (GI_50_) value of ~2 nM and ~70 nM for 4h and 4k, respectively. Across all cell lines tested, a trend towards 4h having higher cellular activity was apparent, but two cell lines stood out as less responsive when compared to all others. In SH-EP cells, which are immortalized but not transformed and express little c-MYC [20], neither CBSI was as effective in comparison to the majority of other cells tested. In addition, in Kelly cells, which harbor two well-characterized genetic mutations (*MYCN* amplification and mutant *ALK*), the response for each CBSI was also reduced. This suggests that the degree of response to 4h and 4k may be based on the mutational status of known cancer drivers.

#### 2.1.2. CBSI Treatment Causes an Increase in Protein Markers of Mitosis

**Figure 2:**
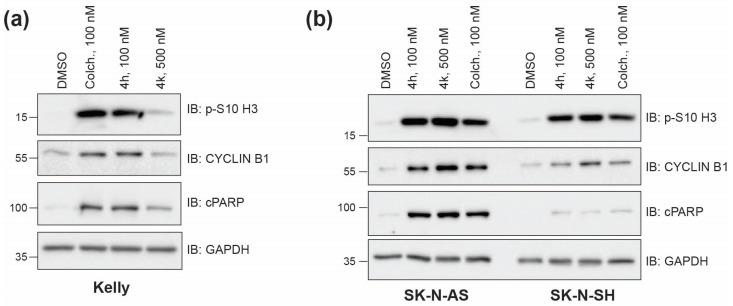
Induction of mitotic and apoptotic protein markers across neuroblastoma cell lines. (**a**) Western blots of protein lysates collected 24 h after treatment of Kelly cells with 0.02% DMSO, 100 nM colchicine, 100 nM 4h, and 500 nM 4k. GAPDH is used as a loading control. P-S10 H3 and CYCLIN B1 are mitotic markers, and cleaved PARP is an apoptotic marker. (**b**) Western blots of protein lysates prepared as in (**a**) from SK-N-AS and SK-N-SH cell lines. Uncropped Western blots used in this figure are included within Supplementary Data, Figure S1.

Tubulin inhibitors should inhibit cell division due to their impact on the active microtubule dynamics required for proper mitosis. To compare the ability of 4h and 4k to act as antimitotic agents in neuroblastoma cells, we assessed the levels of phosphorylated serine-10 on histone 3 (p-S10-H3) or CYCLIN B1 following 24 h incubation with 4h, 4k, or a matched dimethyl sulfoxide (DMSO) control. Because 4h inhibits cell viability at lower concentrations than 4k, we used 100 nM 4h as compared to 500 nM 4k for all treatments. Both p-S10-H3 and CYCLIN B1 levels increase in cells during mitosis and, therefore, each serve as protein markers of response to tubulin inhibition [25,26]. In *MYCN*-amplified Kelly cells, as we saw previously [4], 4h was as effective as commercially available colchicine at increasing the levels of each mitotic marker, while 4k has a moderate effect (Figure 2a). In SK-N-AS and SK-N-SH cells, the level of each mitotic marker was also increased over that of DMSO-treated cells, with compound 4h mirroring the effect of colchicine (Figure 2b). These results suggest that both compounds can cause an accumulation of cells within mitosis, which is indicative of 4h and 4k acting as antimitotic agents. We also probed the levels of cleaved PARP (cPARP), a marker of apoptosis. While all cells tested contain higher levels of cPARP when compared to DMSO-treated cells, there were apparent differences in the level induced (Figure 2a,b), suggesting that CBSI-induced apoptosis may be cell line dependent.

#### 2.1.3. Inhibition of Colony Formation

**Figure 3:**
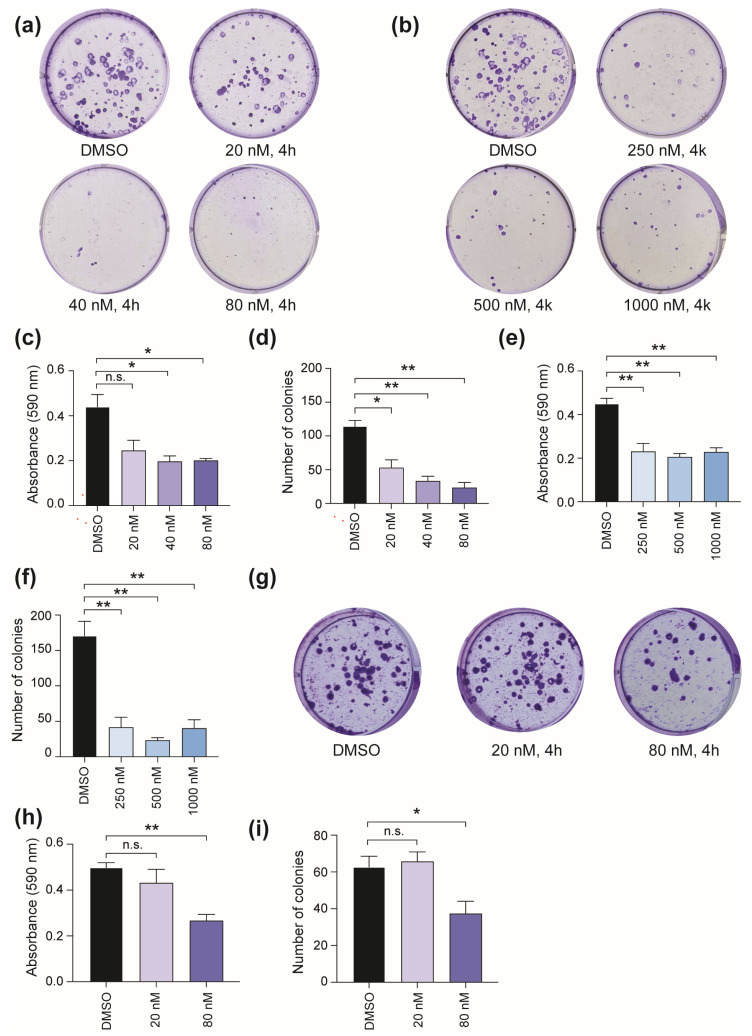
Influence of CBSI treatment on colony formation. (**a**) SK-N-AS cells were treated with 20, 40, and 80 nM 4h or 0.02% DMSO for 24 h, and then compounds were removed. Colonies were allowed to form for 10 days in maintenance media before staining and subsequent analysis. (**b**) SK-N-AS cells were treated and analyzed as in (**a**) except 250, 500, and 1000 nM 4k was used. (**c**) Crystal violet was extracted, and absorbance values were obtained for each condition of 4h treatment. (**d**) Number of colonies were counted for each condition of 4h treatment using ImageJ software version 1.53k. Crystal violet quantification and number of colonies were also determined for 4k treatment and are shown in (**e**) and (**f**), respectively. (**g**) Kelly cells were treated with 20 and 80 nM 4h, or 0.02% DMSO, for 24 h, and then compounds were removed. Colonies were allowed to form for 19 days in maintenance media before staining. (**h**) Crystal violet was extracted, and absorbance values were obtained for Kelly cells treated with indicated doses of 4h. (**i**) Number of colonies were counted for Kelly cells treated with indicated doses of 4h using ImageJ software version 1.53k. (n = 3 biological replicates for each assay shown, error bars are standard error of the mean, ns is not significant, across all data the * *p* < 0.05, and ** *p* < 0.0075, using unpaired Student’s *t*-test, two tailed).

To determine the functional consequence of CBSI treatment, we next asked whether these CBSIs can impair colony formation following 24 h compound treatment. We focused on the *MYCN* single copy cell line, SK-N-AS, which expresses high levels of c-MYC, harbors an 11q deletion risk factor, and has a *RAS* mutation [19,22,23]. The results of the clonogenic assay show that SK-N-AS colony formation is inhibited in a dose-dependent manner upon treatment with 4h (Figure 3a). Compound 4k also impairs colony formation (Figure 3b), although treatment with 4k requires higher doses than 4h based on its lower cellular potency (Table 1). Analysis of crystal violet stain and number of colonies allowed us to confirm that there is a ~50% reduction in colony formation ability achieved using 20 nM 4h (Figure 3c,d) and 250 nM 4k (Figure 3e,f). In comparison, treatment of Kelly cells shows that up to 80 nM 4h is required to achieve a comparable 50% reduction in crystal violet stain and number of colonies (Figure 3g–i). Collectively, these results indicate that while 4h and 4k decrease cell viability (Table 1), and cause enrichment of cells in mitosis as their mechanism of action (Figure 2), the functional consequence of treatment may be dependent on additional factors such as potency of the CBSI, genetic risk factors, and/or the ability of the cells to recover from treatment.

### 2.2. 4h and 4k Reduce Vincristine-Resistant Neuroblastoma Cell Viability in a Dose-Dependent Manner

#### 2.2.1. Cell Viability in Vincristine-Resistant Neuroblastoma Cell Lines

**Figure 4:**
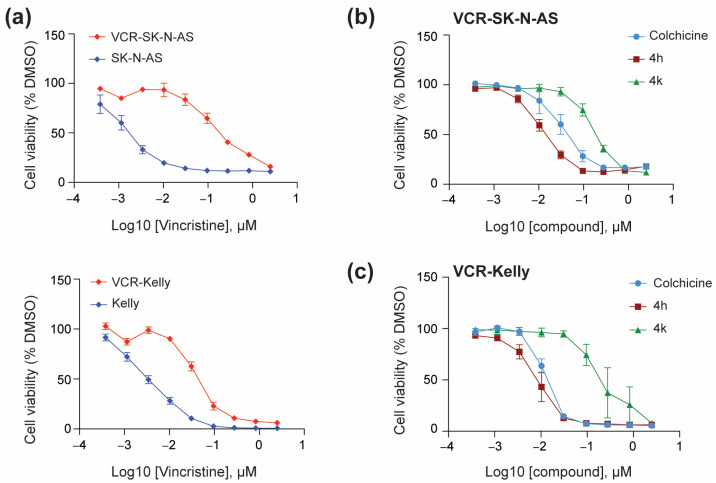
CBSIs are effective against vincristine-resistant neuroblastoma cells. (**a**) SK-N-AS and VCR-SK-N-AS cells (**top**) or Kelly and VCR-Kelly cells (**bottom**) were plated in triplicate and treated with serial dilutions of vincristine or 0.05% DMSO for 72 h before cell viability was assessed using the CellTiter-Glo reagent. Absorbance values were normalized to the DMSO-treated cells before plotting using GraphPad version 10.4.2. (**b**) VCR-SK-N-AS were treated with serial dilutions of colchicine, 4h, and 4k, and then analyzed similarly. (**c**) VCR-Kelly cells were treated and analyzed similarly. (n = 3 biological replicates, error bars are standard error of the mean).

To determine if CBSIs are effective against vincristine-resistant neuroblastoma cell lines, we obtained SK-N-AS and Kelly cells from the Resistant Cancer Cell Line collection [27]. We confirmed that both cell lines are indeed resistant to vincristine as compared to their non-resistant neuroblastoma cell counterparts with vincristine-resistant SK-N-AS (VCR-SK-N-AS) showing a GI_50_ of ~200 nM for vincristine as compared to ~2 nM for SK-N-AS (Figure 4a, top, Table 1). Vincristine-resistant Kelly cells (VCR-Kelly), in comparison, were not as resistant as VCR-SK-N-AS but still show an over 10-fold change in response to vincristine, with VCR-Kelly having a GI_50_ of ~45 nM for vincristine as compared to ~3 nM for Kelly (Figure 4a, bottom, Table 1). We then asked if CBSIs are effective against these vincristine-resistant cells. We observed that colchicine impairs cell viability in both VCR-SK-N-AS (Figure 4b) and VCR-Kelly lines (Figure 4c). Remarkably, 4h is slightly more effective than colchicine, with a cellular potency of ~16 nM in VCR-SK-N-AS cells and ~8 nM in VCR-Kelly cells (Table 1). Again, the cellular potency of 4k is moderate with a GI_50_ of ~200 nM in VCR-SK-N-AS and VCR-Kelly cells (Table 1). However, based on our original cell line paneling data both 4h and 4k remain nearly as effective in vincristine-resistant cell lines as they are in their non-resistant cell counterparts (Table 1), supporting the overall potential of CBSIs to work against cells that have acquired vincristine resistance.

#### 2.2.2. Beta3-Tubulin Expression Levels Do Not Correlate with Cell Line Response

**Figure 5:**
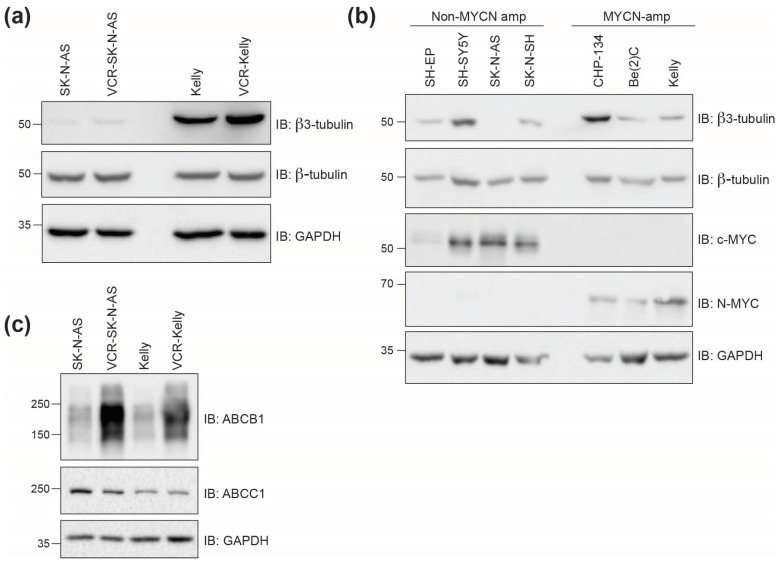
Beta3-tubulin levels do not correlate with *MYCN* amplification status or response to compound treatments. (**a**) Western blots of protein lysates collected from SK-N-AS and Kelly cells compared to lysates from vincristine (VCR)-resistant cells. GAPDH is included as a loading control for comparisons between β-TUBULIN and β3-TUBULIN. (**b**) Western blots of protein lysates from indicated cell lines with varying *MYCN* amplification status. SH-EP, SH-SY5Y, SK-N-AS, and SK-N-SH are non-*MYCN*-amplified, while CHP-134, Be(2)C, and Kelly are *MYCN*-amplified. (**c**) Western blots of protein lysates collected from parental and vincristine-resistant SK-N-AS and Kelly cells showing ABC transporter protein expression. Uncropped Western blots used in this figure are included within the Supplementary Data, Figure S1.

The underlying mechanisms that result in resistance to commonly used clinical tubulin inhibitors such as vincristine (vinca alkaloids) and paclitaxel (taxanes) in the clinic are complex. One common mechanism proposed is altered cellular expression of the Beta3-tubulin isoform, which when incorporated into tubulin dimers can affect tubulin inhibitor function [28]. To determine if the expression of Beta3-tubulin is involved in resistance to vincristine in the neuroblastoma cell lines we tested, we compared the levels of Beta3-tubulin in vincristine-resistant and non-resistant cells. We find that in SK-N-AS cells, Beta3-tubulin levels are minimal, as determined by Western blot, with modest increased expression in VCR-SK-N-AS. In contrast, Kelly cells express high levels of Beta3-tubulin, although it is only moderately increased in the VCR-Kelly line (Figure 5a). Because SK-N-AS and Kelly cells differ in *MYCN*-amplification status, we were curious to understand if *MYCN* expression generally correlates with Beta3-tubulin levels. However, based on Western blot analysis using multiple neuroblastoma cell lines (Figure 5b), we conclude that Beta3-tubulin expression is neither correlated with *MYCN* expression nor with cell line sensitivity to vincristine or CBSI treatment (Table 1). Another common resistance mechanism that can explain resistance to tubulin inhibitors and other chemotherapeutic agents is the overexpression of *ABC* transporters [1,3]. To understand the involvement of ABC transporters in these vincristine-resistant neuroblastoma cells, we probed the expression levels of two well-characterized ABC transporters, ABCC1 and ABCB1. We find that ABCC1 protein levels are similar between resistant and non-resistant cell lines, but that the level of ABCB1 is increased dramatically in both vincristine-resistant cell lines (Figure 5c). While we do not know how ABCB1 is selectively upregulated, we infer based on our cell line paneling data that this increase in ABCB1 does not impede the action of CBSIs as it may for vincristine.

### 2.3. 4h and 4k Treatment Impair Intracellular Microtubule Networks and Act as Antimitotics in Vincristine-Resistant Neuroblastoma Cell Lines

#### 2.3.1. Impact of CBSIs on Intracellular Tubulin and Mitotic Marker Induction

**Figure 6:**
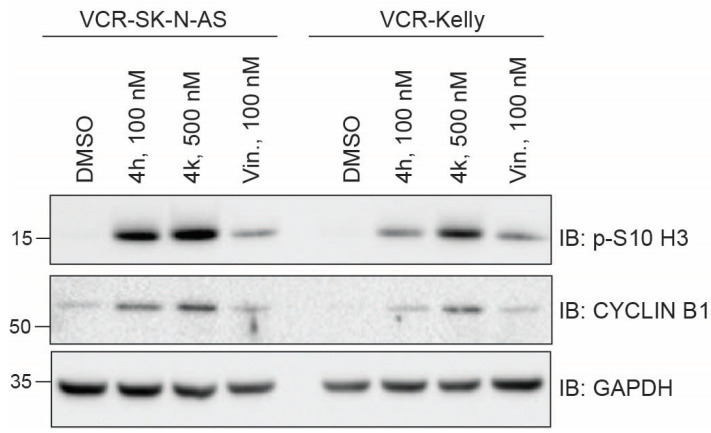
Treatment of vincristine-resistant cell lines with 4h and 4k increases mitotic marker protein levels. Western blots of protein lysates collected 24 h after treatment of VCR-SK-N-AS or VCR-Kelly with 0.02% DMSO, 100 nM 4h, 500 nM 4k, and 100 nM vincristine. GAPDH serves as a loading control. Uncropped Western blots used in this figure are included within the Supplementary Data, Figure S1.

To unravel the primary mechanism of action by which CBSI treatment impairs vincristine-resistant neuroblastoma cell viability, we examined intracellular alpha-tubulin by immunofluorescence in VCR-SK-N-AS and VCR-Kelly cells treated for 24 h with either 100 nM 4h and 500 nM 4k compared to a matched DMSO control. We find that treatment with either 4h or 4k results in the compaction of tubulin around the nuclei in cells imaged within the population (Supplementary Data, Figure S2). This change in morphology is consistent with the morphology of cells in rounded mitosis [29] and indicates that 4h and 4k disrupt microtubule networks within the cells. To begin to assess the influence of 4h and 4k on cell division specifically, we treated resistant cells and examined mitotic marker expression. We included treatment with 100 nM vincristine, which is approaching or above the GI_50_ for each cell line (Table 1), but would allow us a direct comparison between the most active compound, 4h, and vincristine. As anticipated at this dose, 100 nM vincristine causes a moderate increase in the level of p-S10-H3 as compared to DMSO-treated cells, although its impact on increasing CYCLIN B1 is blunted (Figure 6). In contrast, in VCR-SK-N-AS cells, 4h and 4k induce levels of each mitotic marker that are higher than that achieved with DMSO or vincristine treatment. For VCR-Kelly cells, the level of p-S10-H3 and CYCLIN B1 is increased with each compound as compared to DMSO-treated cells, although treatment with 4h more closely mirrors that of vincristine, which we reason is due to VCR-Kelly cells being less vincristine-resistant than VCR-SK-N-AS (Table 1). Regardless, based on these data, we conclude that both 4h and 4k can increase the expression of protein markers of mitosis, suggesting that they cause an enrichment of cells within this phase of the cell cycle.

#### 2.3.2. Cell Cycle Phase Distribution Changes Induced by Treatment with CBSIs

**Figure 7:**
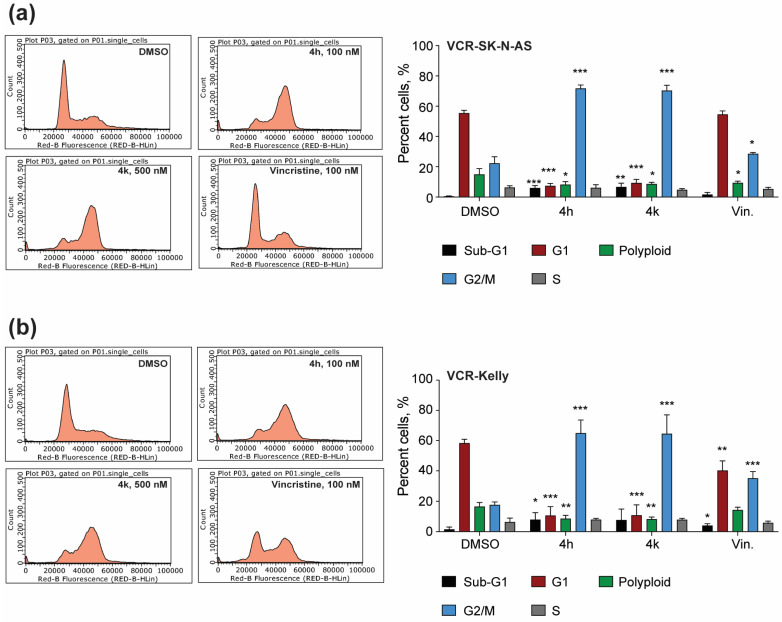
Compounds 4h and 4k impair cell cycle phase distribution in vincristine-resistant neuroblastoma cell lines. (**a**) Representative cell cycle profiles of VCR-SK-N-AS cells treated with 0.02% DMSO, 100 nM 4h, 500 nM 4k, or 100 nM vincristine for 24 h. The percentage of cells in each phase was quantified and reported in the bar graph as shown. (**b**) VCR-Kelly cells were treated as in (**a**) with representative images shown. Quantification of these results can be seen in the associated bar graph. (n = 4 biological replicates, error bars are standard error of the mean, across all data the * *p* < 0.05, ** *p* < 0.005, *** *p* < 0.0004, using unpaired Student’s *t*-test, two tailed).

To directly examine how 4h and 4k impact the number of cells that are in mitosis, we next performed cell cycle phase distribution analysis using flow cytometry. In DMSO-treated VCR-SK-N-AS cells, approximately 20% of the population analyzed is in the G2/M phase (Figure 7a). In contrast, treatment with 4h or 4k for 24 h results in over 60% of cells being in G2/M, which is higher in magnitude than that achieved with vincristine. We also detect significant changes in the percent of cells in the sub-G1, G1, and S phases, indicating that overall 4h and 4k impair VCR-SK-N-AS cell division. In DMSO-treated VCR-Kelly cells, a similar effect was observed, with 4h and 4k causing multiple significant cell cycle phase distribution changes that are more robust than that of vincristine-treated cells (Figure 7b). In sum, mitotic protein marker induction (Figure 6) and cell cycle phase distribution analysis indicate that the primary mechanism of action for CBSIs in these cell lines is to disrupt proper cell division.

### 2.4. Apoptotic Activity of 4h and 4k in Neuroblastoma Cells Compared to Vincristine-Resistant Neuroblastoma Cell Lines

**Figure 8:**
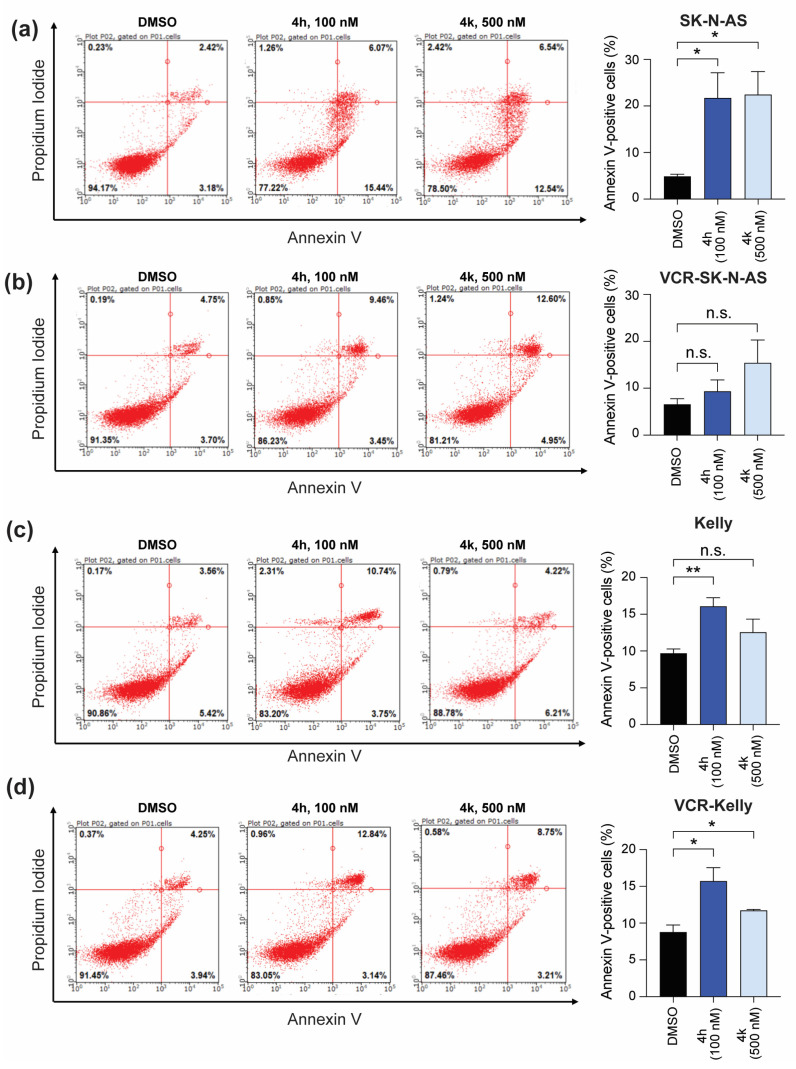
Effect of CBSI treatment on early and late apoptosis. Annexin V staining was performed on SK-N-AS cells (**a**) or VCR-SK-N-AS cells (**b**) with 0.02% DMSO, 100 nM 4h, or 500 nM 4k. The percentages of all Annexin V-positive cells were quantified and reported as bar graphs for each cell line on the right. (**c**) Kelly and (**d**) VCR-Kelly cells were treated similarly and data quantified as shown. (n = 3 biological replicates, error bars are standard error of the mean, ns is not significant, across all data the * *p* < 0.05, ** *p* < 0.008).

Finally, to ask whether inhibition of mitosis by CBSI treatment results in the initiation of apoptosis—and determine any differences in CBSI-induced apoptosis once cells are adapted to vincristine—we performed Annexin V staining followed by flow cytometry. We also co-stained with propidium iodide to capture the number of cells in early and late apoptosis following compound treatment. In SK-N-AS cells, treatment with both 4h and 4k results in a significant increase in the number of Annexin V-positive cells as compared to DMSO-treated controls (Figure 8a). Interestingly, in VCR-SK-N-AS cells, neither 4h nor 4k induce significant changes in Annexin V staining (Figure 8b), indicating that the apoptotic response to CBSI treatment is diminished in these cells. In Kelly cells, both 4h and 4k increase the number of Annexin V-positive cells, although the changes occur in less than 10% of the population and are only significant with 4h treatment (Figure 8c). In contrast to VCR-SK-N-AS cells, the effects of 4h and 4k persist in VCR-Kelly cells, although the magnitude of the effect remains modest (Figure 8d). These results suggest that CBSIs with higher cellular activity are more effective at inducing apoptosis regardless of cell type. In addition, these data reveal that induction of apoptosis is dependent on cell type and most likely cancer cell-specific resistance mechanisms.

## 3. Discussion

The common usage of tubulin inhibitors as a frontline therapy for diverse cancers [30] supports the notion that tubulin inhibitors are a mainstay in cancer treatment. Unfortunately, those that target the vinca alkaloid and taxane binding sites are prone to toxicity-related issues and resistance to treatment is common [6]. In contrast, tubulin inhibitors that target the colchicine binding site show potential in overcoming drug resistance mechanisms, which is a driving force for the continued development of these compounds. A number of CBSIs have entered into clinical trials but as of yet have failed to gain FDA approval. The potent CBSI, verubulin, was unsuccessful in clinical trials due to a number of adverse effects and toxicities observed in patients [31,32]. Plinabulin, on the other hand, has advanced to Phase III clinical trials [33,34] but suffers from poor aqueous solubility thereby limiting its utility [35]. The CBSIs reported in this current study are distinct from verubulin and plinabulin and were discovered as part of a campaign to design ABI-based analogs [36] with restricted conformational flexibility aiming to improve metabolic stability, water solubility, and toxicity profiles while retaining potency [4].

In this study, we performed an in vitro study to investigate the influence of these newly developed CBSIs on neuroblastoma cell function so that we could understand their potential as an anticancer therapy in this cancer context. To understand if CBSIs are broadly effective against neuroblastoma cell lines, we extended our previous cell line paneling [4] to include cell lines marked by genetic risk factors other than *MYCN* amplification. In doing so, we determined that our most potent compound, 4h, is effective against all cell lines tested, although the effect was reduced in two cell lines (SH-EP and Kelly), suggesting the cellular effectiveness of 4h and 4k may be dependent on inherent genetic risk factors. Because SH-EP cells are non-transformed, they are routinely used to express *MYCN* for the study of the encoded N-MYC protein [20]. Because of this, the reduced cellular activity in SH-EP cells compared to other cell lines may indicate a therapeutic window for CBSI treatment, although additional experiments that directly examine the toxicities associated with CBSIs using normal neural crest-derived cells or mouse models would be needed to conclude this. At a mechanistic level, across all cell lines, treatment with 4h and 4k causes accumulation of mitotic protein markers, suggesting that each compound impairs cell division (Figure 2), although a higher concentration of 4k is required to achieve this response as compared to the more potent 4h compound. However, only SK-N-AS cell colony formation was inhibited robustly, and Kelly cells required higher doses to achieve a comparable effect (Figure 3). We cannot rule out that this difference is due to Kelly cells being less responsive to CBSI treatment than SK-N-AS cells (Table 1, Figure 2), but given that the doses we used in the colony formation assay are well above the GI_50_ value for each cell line, we do not think this can fully account for their observed differences. Instead, we think that these findings suggest that the functional consequence of CBSI treatment can vary between cell lines, even though the primary effect of blocking cell division is common. Given that Kelly cells are defined by *MYCN* amplification, it is interesting to speculate on whether *MYCN* status impacts functional response, particularly given the influence of MYC proteins on mitotic cell fate [37]. Further studies that detail the contribution of *MYC* to CBSI response would be very informative to the ~50% of cancers that overexpress this major oncogene [38].

The prevalence of vincristine in current chemotherapy regimens and ongoing clinical trials for neuroblastoma patients [2] prompted us to test the efficacy of CBSIs against vincristine-resistant neuroblastoma cell lines. Notably, we find that 4h, 4k, and colchicine impair vincristine-resistant neuroblastoma cell viability at concentrations similar to those used on non-resistant neuroblastoma cells (Figure 4, Table 1). This finding indicates that CBSIs retain potency even in cells adapted to vincristine. While vincristine-resistant neuroblastoma cells differentially express many genes linked to cancer drug resistance [39], we sought to directly examine two mechanisms of resistance that plague the tubulin inhibitor field—overexpression of the Beta3-tubulin isoform and *ABC* transporters. Expression of Beta3-tubulin occurs often in cancer, correlates with poor outcomes, and results in resistance to taxane-based tubulin inhibition (i.e., paclitaxel) [28]. As it relates to vinca alkaloid resistance, the opposite may be true with decreased levels of the isoform correlating with increased microtubule stability and resistance [40]. Importantly, overexpression of *ABC* transporters can lead to resistance to both subtypes of clinically used tubulin inhibitors [3]. To determine any relationship between beta3-tubulin, ABC transporters, and compound response in the cells used in our study, we assessed protein expression in non-resistant and resistant cell lines (Figure 5). While we did not observe any correlation between the levels of beta3-tubulin and response to vincristine or CBSI treatment, we did notice that expression of ABCB1 is highly overexpressed in vincristine-resistant cells. These data imply that ABCB1 expression is one factor that correlates with resistance to vincristine, but based on our cell line paneling data (Table 1), its increased levels do not severely limit the efficacy of CBSIs tested here.

While we may not fully understand all factors that predict overall response to vincristine or CBSIs, what we do find here is that 4h and 4k both act against vincristine-resistant cells through a similar mechanism centered on blocking the ability of cells to complete cell division through mitosis (Figure 6 and Figure 7). This supports the effectiveness of CBSIs as antimitotic agents for treatment against primary and resistant cancers. When we tested the ability of compound treatment to induce apoptosis, we observed that 4h, and to a lesser extent, 4k, is capable of inducing apoptosis (Figure 8), pointing to 4h being superior to 4k in inducing cell death. Neither Kelly nor SK-N-AS cells express functional p53 [41,42], so we deduce that 4h and 4k promote apoptosis through a p53-independent pathway, which is not uncommon for tubulin inhibitors used in other cancers [43,44]. We therefore suspect that the level of apoptosis we see is an underestimate of what these CBSIs could achieve in wild-type p53 cells, which is important to take into consideration due to the fact that neuroblastomas rarely contain *TP53* mutations at the time of diagnosis [45]. Intriguingly, all apoptotic response to CBSI treatment is lost in VCR-SK-N-AS cells but maintained in VCR-Kelly cells, suggesting that distinct resistance mechanisms can emerge when neuroblastoma cells become adapted to vincristine. Based on these data and that of others in the field, we think that at least part of the differential response involves altered regulation of pro- and anti-apoptotic protein expression [40,46], although it is also possible that the maintained apoptotic response we observe in VCR-Kelly cells relates to the ability of MYC proteins to sensitize cells to tubulin inhibitor treatment [37].

In sum, our study has provided a solid foundation for investigating CBSIs as a treatment approach for neuroblastoma, especially as progress is made to increase the solubility, metabolic stability, and therapeutic window of these agents. However, it is important to keep in mind that the data collected here use a series of in vitro biological assays and are limited to one type of therapy used in neuroblastoma treatment. As such, advanced studies will be necessary to challenge the utility of CBSIs as improved chemotherapeutics. Future development of compounds like 4h and 4k may benefit from testing in vincristine-resistant cell lines so that the most broadly effective inhibitors can be identified. In addition, efforts in finding drug combinations that shift the response of CBSIs to one that is profoundly cytotoxic may also be of value as the CBSIs we tested impair cell viability through a mixture of cytostatic and cytotoxic effects. Combination approaches have shown promise in progressive neuroblastoma and diffuse large B-cell lymphoma to promote vincristine response [46,47], suggesting similar strategies could be employed with CBSIs as well. Experiments such as those described above—or others that probe the benefits and limitations of this new family of tubulin inhibitors—should be illuminating for the treatment of cancer.

## 4. Materials and Methods

### 4.1. Cell Lines

Vincristine-resistant Kelly and SK-N-AS cells were obtained from the Resistant Cancer Cell Line collection (Canterbury, UK) [27]. They were maintained in IMDM media supplemented with 10% fetal bovine serum (FBS), 1% Penicillin/Streptomycin, and 10 nM of vincristine. Non-resistant SK-N-AS, SH-EP, SH-SY5Y, SK-N-SH, and IMR-32 were provided by Dr. Dai Chung. SH-EP cells used in cell line paneling were provided by Dr. Rogier Versteeg and are the SH-EP P2 control cell line used for studying the overexpression of *MYCN*. Kelly and CHP-134 cells were obtained from Sigma (St. Louis, MO, USA). These cell lines were maintained in RPMI-1640 media with l-glutamine containing 10% FBS and 1% Penicillin/Streptomycin.

### 4.2. Protein Lysates and Western Blotting

Approximately two million cells were harvested per protein lysate. For compound treatments, lysates were generated following a 24 h treatment. All cells were lysed in cold lysis buffer containing 150 mM Tris, pH 8.0, 5 mM EDTA, 150 mM NaCl, 1% Triton X-100, PMSF, and Roche protease inhibitor cocktail through sonication for 15 s at 25% power. Lysates were centrifuged to remove debris before protein concentrations were determined using the BioRad Bradford assay. Proteins were resolved using SDS-PAGE, transferred to a PVDF membrane (Pierce, Rockford, IL, USA), and membranes blocked with a 5% nonfat dry milk blocking solution made in TBS-T (50 mM Tris, pH 7.5, 0.1% Tween-20, 150 mM NaCl). Immunoblotting was performed using primary antibodies for each respective protein. The antibodies used were as follows: c-MYC (Abcam, Cambridge, UK, AB32072), N-MYC (Cell Signaling, Waltham, MA, USA, 51705), GAPDH-HRP (Cell Signaling, 8884), p-S10-H3 (Cell Signaling, 53348), β-TUBULIN (Cell Signaling, 2128), β3-TUBULIN (Cell Signaling, 5568), cleaved PARP (Cell Signaling, 9541), CYCLIN B1 (Cell Signaling, 4138), ABCB1 (Cell Signaling, 13342), and ABCC1 (Cell Signaling, 72202). Band visualization was performed using the Clarity ECL substrate (BioRad, Hercules, CA, USA) and a BioRad ChemiDoc MP instrument.

### 4.3. Colony Formation Assay

Kelly and SK-N-AS cells were seeded in 6-well plates at a density of 400 and 800 cells per well, respectively. Following incubation for 24 h in varying concentrations of the compounds or DMSO, the media was aspirated and replenished with normal maintenance media. Cells were left to grow for an additional 9–18 days and then they were stained with a 0.5% crystal violet solution made of fresh ice-cold 70% methanol. After washing with deionized water, colonies were imaged and crystal violet was quantified after extraction from each sample using 33% acetic acid. The absorbance of extracted crystal violet was read in triplicate using a Clariostar plate reader (BMG LABTECH, Ortenberg, Germany) set at an absorbance of 590 nm.

### 4.4. Cell Viability Screening

To assess cell viability response due to compound treatment, 5000 cells per cell line were plated in triplicate on a 96-well plate using 0.05% DMSO or a nine-point serial dilution of compounds. Commercially available colchicine (MedChem Express, Monmouth Junction, NJ, USA) or vincristine (Selleck Chemical, Houston, TX, USA) were included depending on the experiment performed. After three days of incubation, the samples were processed using the CellTiter-Glo assay reagent (Promega, Madison, WI, USA). In brief, 50 μL of CellTiter-Glo was added to each well and incubated at room temperature for 30 min before being placed on a Clariostar plate reader, which determines luminescence values. GraphPad Prism version 10.4.2 was used to calculate GI_50_ values.

### 4.5. Cell Cycle Analysis

The 2.0 × 10^6^ vincristine-resistant Kelly or SK-N-AS cells were plated equally with media containing either 100 nM 4h, 500 nM 4k, 100 nM vincristine, or 0.02% DMSO. After a 24 h incubation period, 1.0 × 10^6^ cells were collected and fixed in ice-cold 70% ethanol. Fixed cells were then stored at −20 °C until ready to stain. For staining, cells were allowed to thaw at room temperature before being washed in phosphate-buffered saline (PBS). After washing, cells were resuspended in a propidium iodide (PI) staining solution (PBS, 10 μg/mL PI, 100 μg/mL RNAse A inhibitor, 2 mM MgCl_2_) before being incubated overnight at 4 °C. The next day, the stained cells were filtered through a 35 μm nylon mesh cell strainer. A minimum of 10,000 single cells from each cell line was counted using a Guava easyCyte Flow Cytometer (Luminex, Austin, TX, USA) with selection for single cells based on forward and side scatter.

### 4.6. Annexin V Staining

Annexin staining was completed using the Dead Cell Apoptosis Kit with Annexin V Alexa Fluor 488 and PI (V13241, ThermoFisher, Waltham, MA, USA). 2.0 × 10^6^ cells were treated with compounds or controls. For incubation, Kelly lines were left for 24 h and SK-N-AS lines for 48 h. After incubation, 1.0 × 10^6^ cells were rinsed in cold PBS before being centrifuged at 900 RPM for 5 min. Cells were resuspended in an annexin-binding buffer, and 5 μL of Alexa Fluor 488 and 1 μL of 100 μg/mL PI solution (Annexin-binding buffer, 1 mg/mL PI stock solution), followed by incubation for 15 min. After incubation, samples were diluted in 400 μL of annexin-binding buffer and filtered through a 35 μm nylon mesh cell strainer followed by immediate analysis by flow cytometry. The number of cells showing PI (red) or Alexa Fluor 488 (green) fluorescence was analyzed using a Guava easyCyte Flow Cytometer (Luminex) with selection for single cells based on forward and side scatter and gating against the DMSO-treated sample. At least 7000 single cells were recorded for each sample.

### 4.7. Immunofluorescence

Resistant cell lines were plated on coverslips coated with 0.1 mg/mL poly-D-lysine (Gibco, Billings, MT, USA). Cells were treated with the indicated doses of compounds for 24 h and then fixed with 4% methanol-free formaldehyde. Cells were permeablized in 0.2% Triton X-100 for ten minutes and blocked in a solution containing 5% goat serum and 3% bovine serum albumin in PBS for a minimum of 1 h prior to adding a primary antibody against alpha-tubulin (1:100, Proteintech, Rosemont, IL, USA, 11224-1-AP) overnight at 4 °C. The next day, coverslips were washed three times with PBS before an Alexa-488 goat anti-rabbit antibody was added (1:500, Invitrogen, Carlsbad, CA, USA, A11008) for 1 h at room temperature. Following an additional three washes, coverslips were mounted on slides using ProLong Diamond Antifade Mounting media containing DAPI (Invitrogen, P36966). Approximately 10–15 images were obtained per sample at 60X using an Olympus IX83 instrument (West Chester, PA, USA). ImageJ software version 1.53k was used to generate composite images, as shown in Supplementary Data Figure S2.

## Figures and Tables

**Table 1:** Cell viability results for all cell lines used in this study. Cell lines were treated for three days with nine serial dilutions of indicated compounds or dimethyl sulfoxide (DMSO) as a control. Cell viability values obtained were normalized to those from dimethyl-sulfoxide (DMSO)-treated wells and GI_50_ values were calculated for all compounds (n = 3 biological replicates, data show GI_50_ value with standard error of the mean). Information on genetic factors for each cell line was obtained from [19,20,21,22,23,24]. SH-EP cells are considered non-transformed but immortalized and express very low levels of c-MYC with no other notable genetic lesions [20]. “VCR” = vincristine-resistant, “NT” = not tested.

Cell Line	*MYCN* Status	Genetic Risk Factor	4h (GI_50_, nM)	4k (GI_50_, nM)	Vincristine
SK-N-SH	Single copy	* ALK * ^ F1174L ^	1.05 ± 0.15	55.23 ± 10.34	NT
SK-N-AS	Single copy	11q deletion, *NRAS*^Q61K^	1.02 ± 0.28	98.63 ± 12.75	1.83 ± 0.28
SH-SY5Y	Single copy	*ALK* ^F1174L^	0.95 ± 0.17	59.15 ± 12.23	NT
SH-EP	Single copy	N/A	12.92 ± 4.38	389.80 ± 75.21	NT
IMR-32	Amplified	*MYCN* amp	1.57 ± 0.36	69.13 ± 12.10	NT
Kelly	Amplified	*MYCN* amp, *ALK*^F1174L^	13.90 ± 2.32	300.00 ± 93.40	3.44 ± 0.21
CHP-134	Amplified	*MYCN* amp	1.72 ± 0.28	45.19 ± 8.71	NT
VCR-SK-N-AS	Single copy	11q deletion, *NRAS*^Q61K^	15.90 ± 2.16	203.00 ± 20.40	206.00 ± 26.80
VCR-Kelly	Amplified	*MYCN* amp, *ALK*^F1174L^	8.30 ± 1.12	233.00 ± 55.90	45.40 ± 5.14

## Data Availability

The raw data supporting the conclusions of this article will be made available by the authors upon request.

## Supplementary Materials

The following supporting information can be downloaded at: https://www.mdpi.com/article/10.3390/molecules30102186/s1, Figure S1: Uncropped Western blots. The images shown are associated with Figure 2, Figure 5, and Figure 6 in the main text. It includes the uncropped Westerns displayed in this study, along with merged images that include molecular weight markers. Figure S2: Distribution of intracellular ⍺-tubulin in vincristine-resistant cell lines following compound treatment. Vincristine-resistant Kelly or SK-N-AS cells were treated with 100 nM 4h, 500 nM 4k, or a matched DMSO control for 24 h. Representative images show ⍺-tubulin in green and nuclei in blue (scale bar = 20 μm, n = 3 biological replicates).

## Abbreviations

The following abbreviations are used in this manuscript:

VCR	Vincristine-resistant
CBSI	Colchicine binding site inhibitor
DMSO	Dimethyl sulfoxide
ABC	ATP-binding cassette
