# Supplementary material for: Colchicine Binding Site Tubulin Inhibitors Impair Vincristine-Resistant Neuroblastoma Cell Function
Source: Molecules. 2025 May 16;30(10):2186. doi: 10.3390/molecules30102186 (PMC12113804; doi:10.3390/molecules30102186)
Supplement: Supplementary file 1 [file molecules-30-02186-s001.zip › molecules-3582832-supplementary.pdf]

Supplementary Materials

Colchicine binding site tubulin inhibitors impair vincristine-resistant neuroblastoma cell function

Reed, C.N. et al.

Included figures:

Figure S1. Uncropped Western blots. The images shown are associated with Figures 2, 5, and 6 in the main text. It includes the uncropped Westerns displayed in this study, along with merged images that include molecular weight markers.

Figure S2. Distribution of intracellular α-tubulin in vincristine-resistant cell lines following compound treatment. Vincristine-resistant Kelly or SK-N-AS cells were treated with 100 nM 4h, 500 nM 4k, or a matched DMSO control for 24 hr. Representative images show α-tubulin in green and nuclei in blue (scale bar = 20 μm, n = 3 biological replicates).

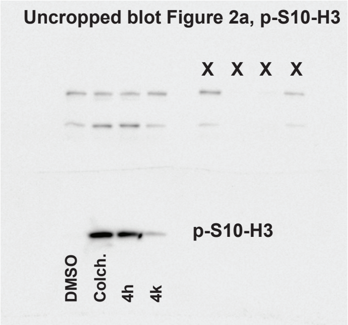

Uncropped blot Figure 2a, p-S10-H3

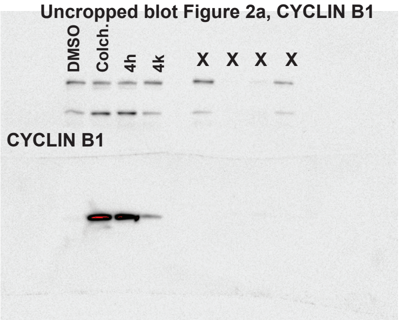

Uncropped blot Figure 2a, CYCLIN B1

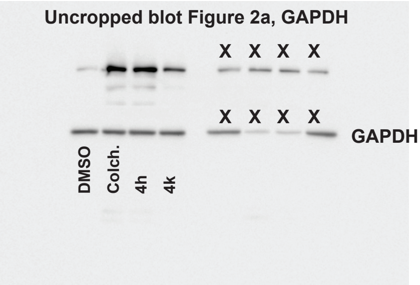

Uncropped blot Figure 2a, GAPDH

Darker uncropped blot Figure 2a with Ladder merge for p-S10-H3 and CYCLIN B1 antibody

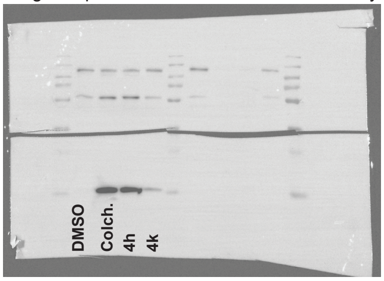

Darker uncropped blot Figure 2a with Ladder merge for cPARP and GAPDH antibody

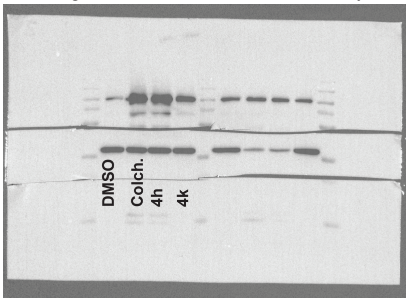

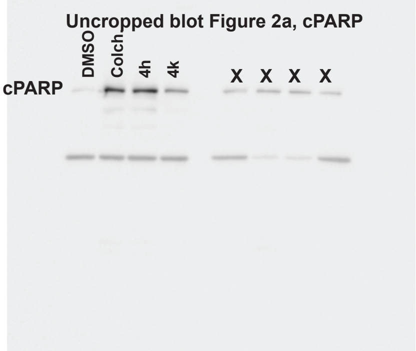

Uncropped blot Figure 2a, cPARP

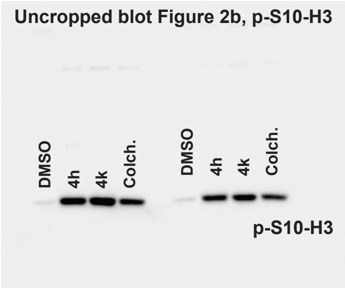

Uncropped blot Figure 2b, p-S10-H3

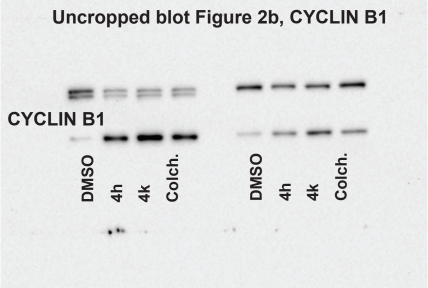

Uncropped blot Figure 2b, CYCLIN B1

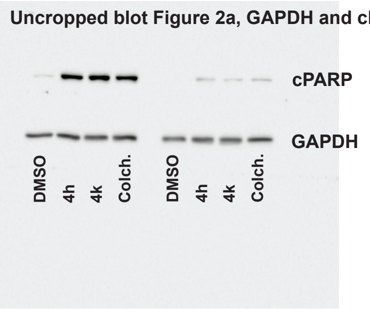

Uncropped blot Figure 2a, GAPDH and cPARP

Uncropped blot Figure 2b with Ladder merge for p-S10-H3 antibody

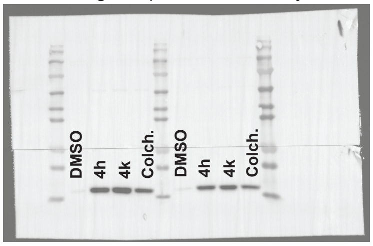

Uncropped blot Figure 2b with Ladder merge for CYCLIN B1 antibody

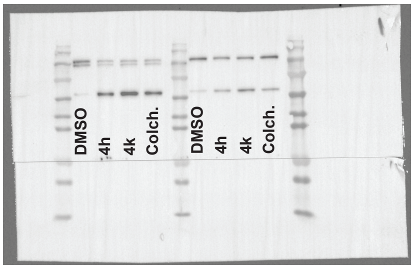

Uncropped blot Figure 2b with Ladder merge for cPARP and GAPDH antibody

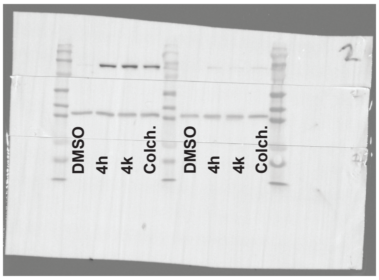

Uncropped blot Figure 5a, Beta3-tubulin

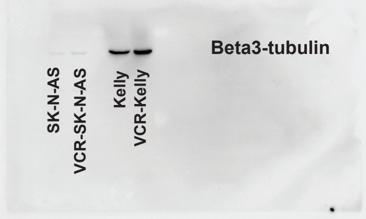

Uncropped blot Figure 5a, GAPDH

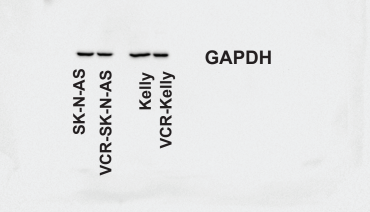

Uncropped blot Figure 5a, Beta-tubulin

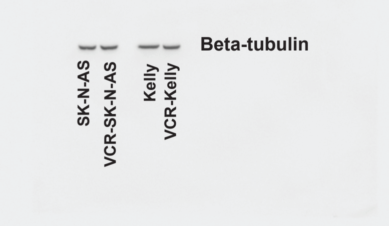

Uncropped Figure 5a with Ladder merge for beta3-tubulin antibody

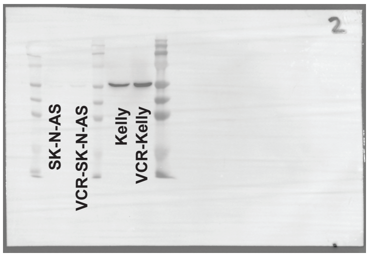

Uncropped blot Figure 5a with Ladder merge for GAPDH antibody

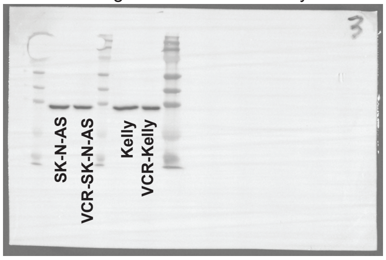

Uncropped blot Figure 5a with Ladder merge for beta-tubulin antibody

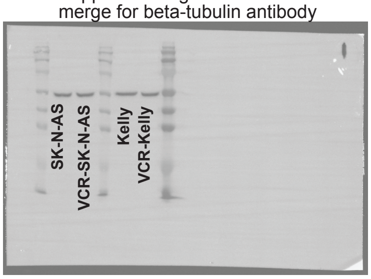

Uncropped blot Figure 5b, Beta3-tubulin

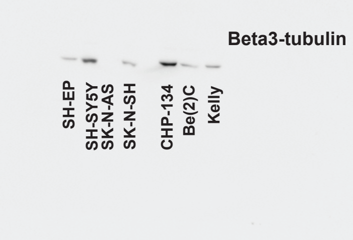

Uncropped blot Figure 5b, c-MYC

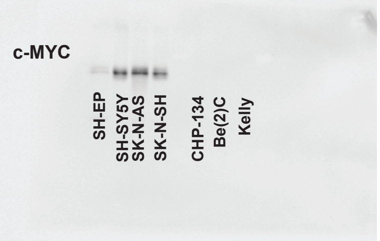

Uncropped blot Figure 5b, N-MYC

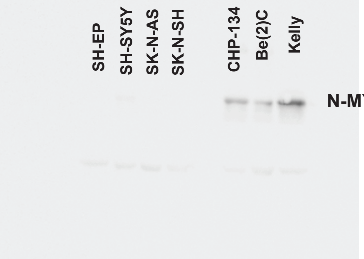

Uncropped blot Figure 5b, Beta-tubulin

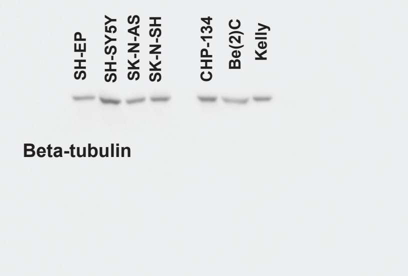

Uncropped blot Figure 5b, GAPDH

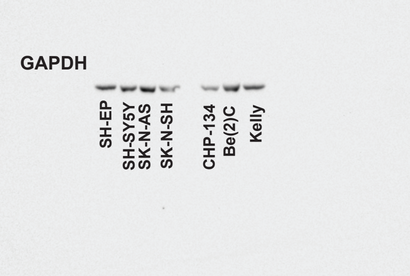

Uncropped Figure 5b with Ladder merge for beta3-tubulin antibody

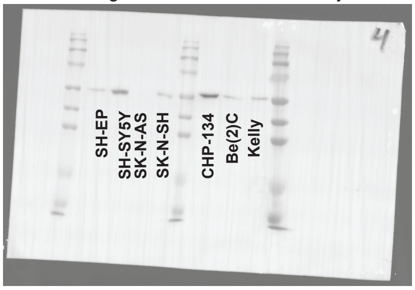

Uncropped Figure 5b with Ladder merge for c-MYC antibody

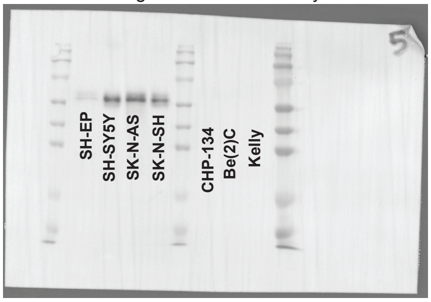

Uncropped Figure 5b with Ladder merge for N-MYC antibody

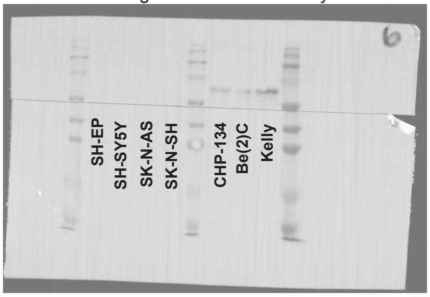

Uncropped Figure 5b with Ladder merge for beta-tubulin antibody

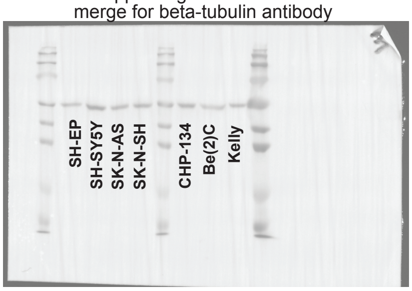

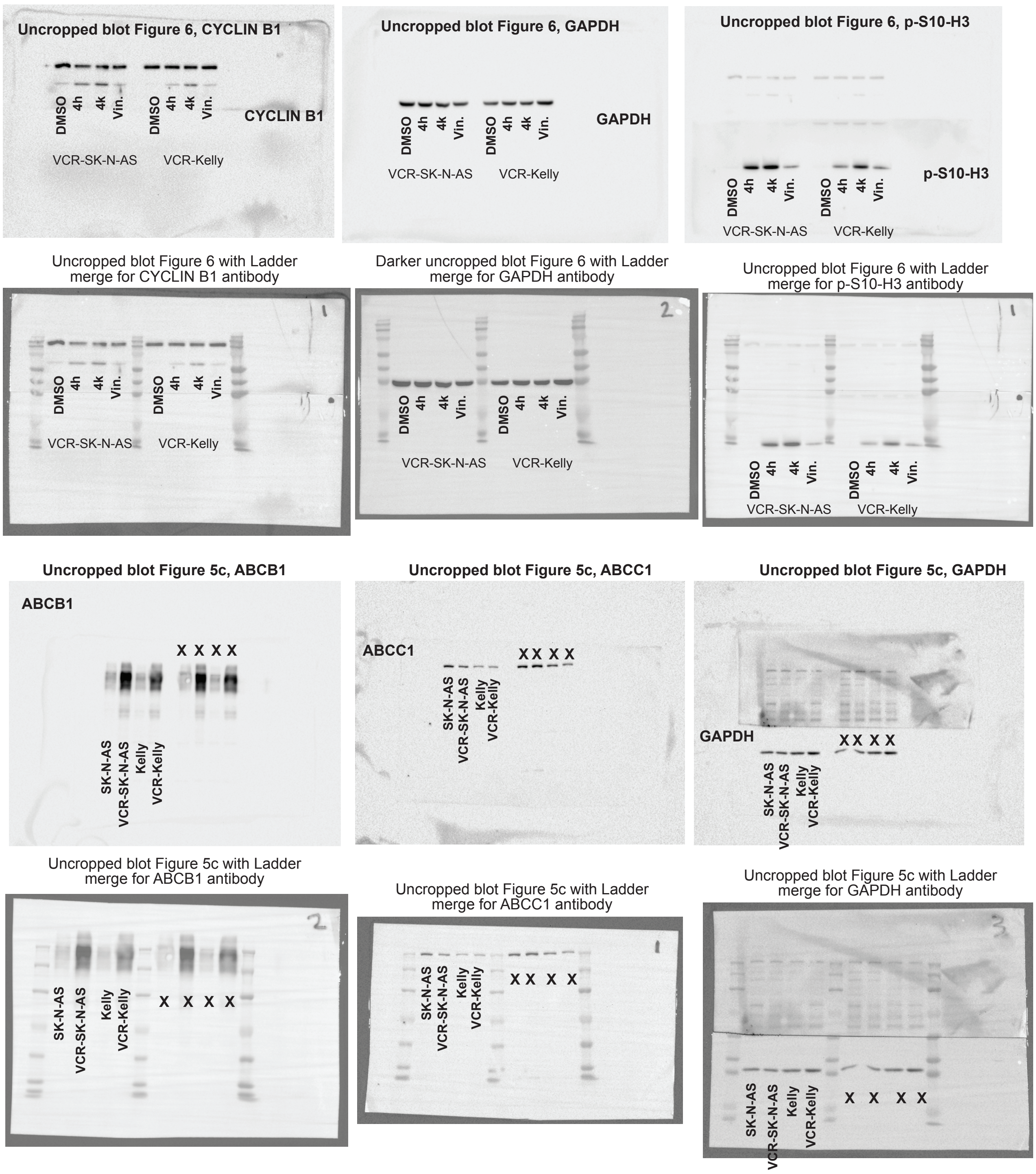

Figure S1. Uncropped Western blots. The images shown are associated with Figures 2, 5, and 6 in the main text. It includes the uncropped Westerns displayed in this study, along with merged images that include molecular weight markers.

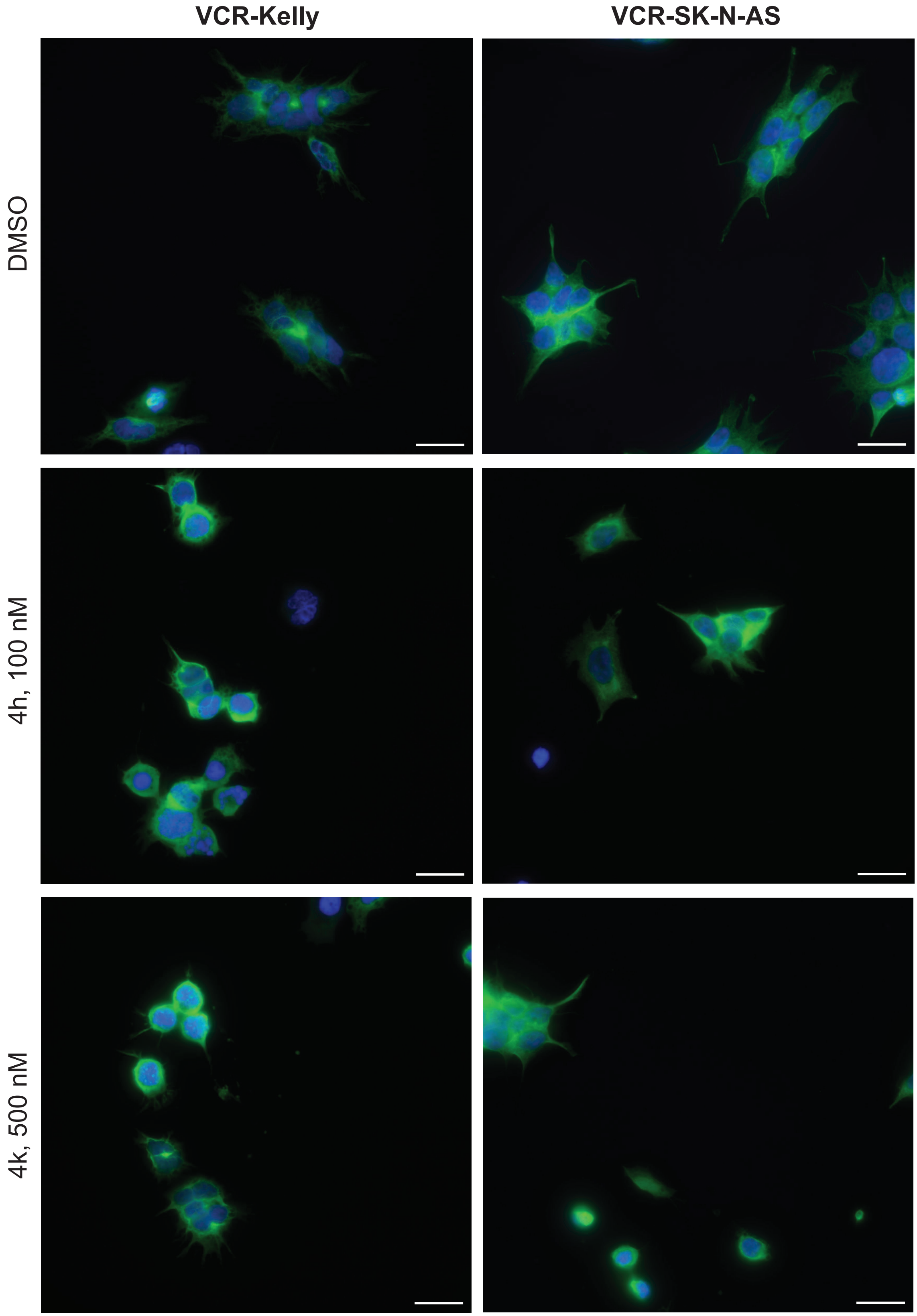

VCR-Kelly
VCR-SK-N-AS
DMSO
4h, 100 nM
4k, 500 nM

Figure S2. Distribution of intracellular α-tubulin in vincristine-resistant cell lines following compound treatment. Vincristine-resistant Kelly or SK-N-AS cells were treated with 100 nM 4h, 500 nM 4k, or a matched DMSO control for 24 hr. Representative images show α-tubulin in green and nuclei in blue. The scale bar = 20 μm, n = 3 biological replicates).
